# Complete genome sequences of two biocontrol *Bacillus velezensis* strains isolated from soil in Japan

**DOI:** 10.1128/mra.00509-26

**Published:** 2026-07-10

**Authors:** Ruka Izawa, Nobutaka Someya, Hiroki Nakahara, Masaharu Kubota, Tomohiro Morohoshi

**Affiliations:** 1 Graduate School of Regional Development and Creativity, Utsunomiya University 13144, Utsunomiya, Tochigi, Japan; 2 Institute for Plant Protection, National Agriculture and Food Research Organization (NARO) 13516, Tsukuba, Ibaraki, Japan; Wellesley College, Wellesley, Massachusetts, USA

**Keywords:** *Bacillus velezensis*, biocontrol, complete genome sequence

## Abstract

Here, we report the complete genome sequences of two strains of *Bacillus velezensis*, S1022 and S1172, isolated from soil in Japan. The genome sizes of S1022 and S1172 were 4,020,394 bp with a GC content of 46.4% and 3,995,622 bp with a GC content of 46.5%, respectively.

## ANNOUNCEMENT

*Bacillus velezensis* is a well-known biocontrol agent against soil-borne plant pathogens (1). We isolated spore-forming bacterial strains from soil samples in Japan (2) by heat treatment followed by cultivation on nutrient agar plates at 25°C for 72 h. Two strains, S1022 and S1172, were isolated in Kagoshima, Japan (2), and their 16S ribosomal RNA (rRNA) sequences were amplified using primers 10F (5′-GTTTGATCCTGGCTCA-3′) and 800R (5′-TACCAGGGTATCTAATCC-3′) (3) (Bacterial 16S rDNA PCR Kit Fast (800); Takara Bio, Shiga, Japan) and showed high identity (>99%) with *B. velezensis* NRRL B-41580^T^ (GenBank accession no. KY694464). Preliminary studies showed that S1022 and S1172 exhibit antagonistic activities against fungal plant pathogens in dual culture assays, with fungal plugs showing reduced mycelial growth compared to controls. Genome sequencing was performed to elucidate the genetic basis of antimicrobial activity and biocontrol potential. We obtained and analyzed the genomes of strains S1022 and S1172 to examine biocontrol activity.

Single colonies of both strains were formed on Tryptic Soy Broth (TSB; Becton, Dickinson and Co., Sparks, MD, USA) agar plates from glycerol stocks, inoculated into TSB liquid medium using sterile toothpicks, and aerobically incubated overnight at 30°C with shaking. Bacterial cells were collected by centrifugation, and genomic DNA was extracted using NucleoBond HMW DNA (Takara Bio) according to the manufacturer’s instructions. Library construction and sequencing were performed using the commercial services of Bioengineering Lab. Co., Ltd. (Kanagawa, Japan). Genomic DNA was sheared to approximately 10–25 kb, and a DNA library was constructed using the SMRTbell Prep Kit 3.0 and SMRTbell gDNA Sample Amplification Kit (Pacific Biosciences, Menlo Park, CA, USA). Sequencing was performed using a PacBio Revio sequencer (Pacific Biosciences) with the Revio Polymerase Kit according to the manufacturer’s instructions. Overhang adapter sequences were removed using SMRT Link v13.0.0.207600 to obtain subreads. After aligning these subreads to create a consensus sequence, consensus reads with an average quality score per read below 20 were removed, resulting in high-fidelity (HiFi) reads. PCR adapters, PCR duplicates, and reads shorter than 1,000 bases were removed using lima v2.7.1, pbmarkdup v1.0.3, and Filtlong v0.2.1. The sequencing reads were assembled using Flye v2.9.6 (4). The assemblies were annotated using the DNA Data Bank of Japan (DDBJ) Fast Annotation and Submission Tool (DFAST) pipeline v1.2.16 (5). The coding sequences (CDSs) were predicted using MetaGeneAnnotator v2008/08/19 (6). Genes encoding transfer RNA (tRNA) and rRNA were identified using Aragorn v1.2.38 (7) and Barrnap v0.8 (https://github.com/tseemann/barrnap), respectively. MetaGeneAnnotator, Aragorn, and Barrnap were selected as parameters within the DFAST pipeline. Default parameters were used for all software, unless otherwise specified.

The genome of S1022 consists of a single chromosome, whereas S1172 has a single chromosome and an endogenous plasmid (Table 1). Flye assembly generated single circular contigs for both replicons without gaps, with circularization confirmed by the assembler. Whole-genome similarity to *B. velezensis* NRRL B-41580^T^ was evaluated by average nucleotide identity (ANI) and digital DNA-DNA hybridization (dDDH). ANI and dDDH values were calculated using the OrthoANIu tool (https://www.ezbiocloud.net/tools/ani) and Genome-to-Genome Distance Calculator v3.0 (formula 2) (8, 9), both exceeding species thresholds (Table 1), classifying them as *B. velezensis*.

**TABLE 1:** Genomic information of the two strains of *B. velezensis* isolated from soil in Japan

Genome characteristics	S1022	S1172
Number of PacBio HiFi reads	37,051	31,636
Total bases (bp)	247,933,985	249,079,421
PacBio read N50 (bp)	7,510	9,110
Genome size (bp)	4,020,394	3,995,622
Chromosome size (bp)	4,020,394	3,979,770
Plasmid size (bp)	–^*a*^	15,852
Coverage (×)	61.7	62.3
CDSs	3,819	3,839
rRNA	27	27
tRNA	86	86
GC content (%)	46.4	46.5
BioProject accession no.	PRJDB42055	PRJDB42055
BioSample accession no.	SAMD01899375	SAMD01899376
DRA accession no.	DRR959715	DRR959716
DDBJ/ENA/GenBank accession no.	AP046636	AP046637, AP046638
ANI value with NRRL B-41580^T^ (%)	98.15	97.72
dDDH value with NRRL B-41580^T^ (%)	84.0	79.2

^*a*^ –, does not have any plasmids.

## Data Availability

The genome sequences have been deposited in the DNA Data Bank of Japan (DDBJ) under accession numbers AP046636 (strain S1022) and AP046637–AP046638 (strain S1172). The raw sequence reads have been deposited in the DDBJ Sequence Read Archive (DRA) under accession numbers DRR959715 (strain S1022) and DRR959716 (strain S1172).
